# Supplementary material for: Rational design of a survey protocol for avocado sunblotch viroid in commercial orchards to demonstrate pest freedom
Source: PLoS One. 2023 Apr 11;18(4):e0277725. doi: 10.1371/journal.pone.0277725 (PMC10089318; doi:10.1371/journal.pone.0277725)
Supplement: S1 File — (PDF) [file pone.0277725.s001.pdf]

S1 Additional figures, mathematical formulas and interactive computation tool

A. Supplementary figures

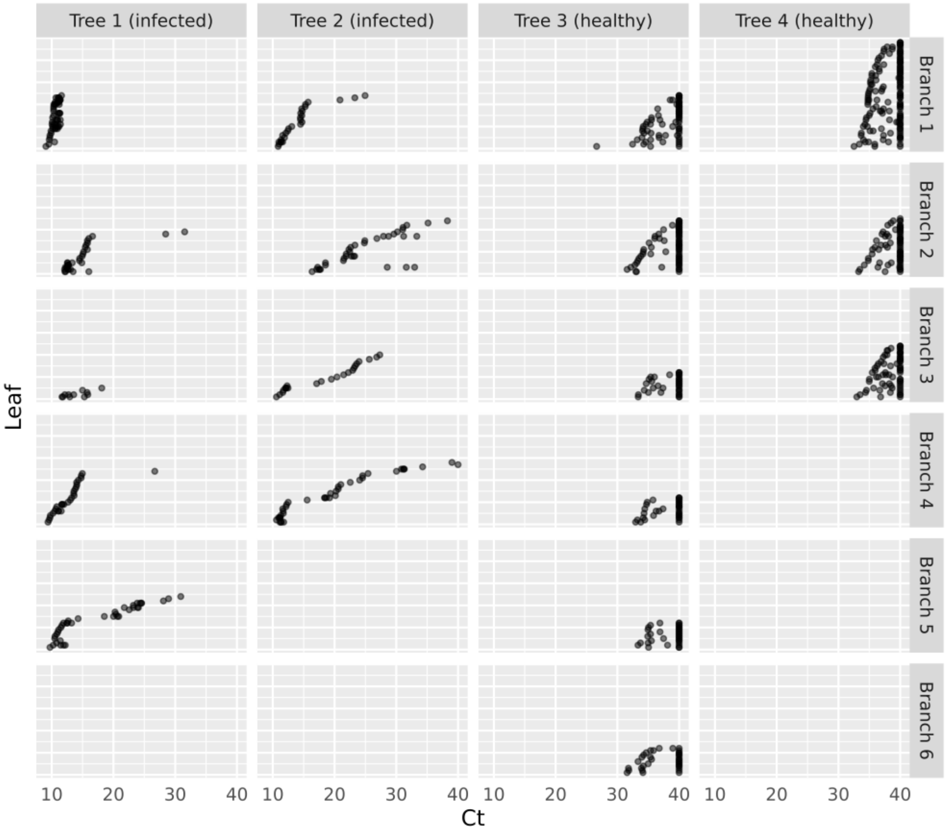

S1 Figure 1 Ct measurements by tree, branch, leaf, biological replicate index.

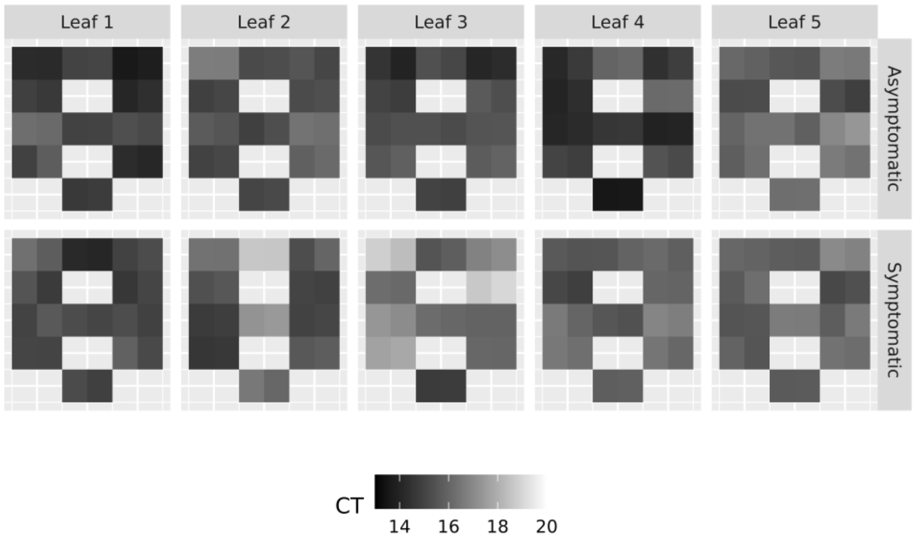

S1 Figure 2. Heatmaps for five asymptomatic and five symptomatic leaves.

B. Hierarchical modelling for Ct measurements

B.1. General model

Equation (S1 Eqn 1) with a corresponding model and in Equation (S1 Eqn 2) where leaf samples were collected from octants instead of randomly selected branches.

$$Ct_{tree\,status,tree,branch,leaf,disc,technical\,replicate} = \mu_{tree\,status} + \varepsilon_{tree} + \varepsilon_{tree.branch} + \varepsilon_{leaf} + \varepsilon_{leaf,disc} + \varepsilon_{disc} + \varepsilon_{technical\,replicate} \quad \text{(S1 Eqn 1).}$$

$$Ct_{tree\,status,tree,octant,leaf,disc,technical\,replicate} = \mu_{tree\,status} + \varepsilon_{tree} + \varepsilon_{tree,octant} + \varepsilon_{octant} + \varepsilon_{leaf} + \varepsilon_{leaf,disc} + \varepsilon_{disc} + \varepsilon_{technical\,replicate} \quad \text{(S1 Eqn 2).}$$

Under the models in Equations (S1 Eqn 1) and (S1 Eqn 2), Ct measurements for leaves of an infected or healthy tree (ASBVd tree status) are the sums of a theoretical average Ct measurement and variations due to the tree branch (or octant), the leaf, the sampling location in the leaf and random noise associated with the RT-qPCR assay.

B.2. Experiment 1

We tested the (nested or crossed) leaf, branch, (nested or crossed) octant, and tree effects. Before running the mixed model analysis, the assumption of homogeneity of variance of the Ct values was tested and variance shown to be equal across the samples (Bartlett test, p. value: 0.47). There was evidence of a fixed effect of the branch (LRT, p-value<0.0001), branch as well as nested octant (LRT, p-value<0.0001), of a leaf random effect (LRT, p-value<0.0001) and biological replicate (LRT, p-value<0.0001). The assumption of a crossed versus a nested fixed effect of octant (LRT, p-value<0.0001) was rejected.

B.3. Experiment 2

The statistical aim of Experiment 2 was to test the effect of the position of the leaf disc, to estimate the variance between leaf discs of the mixed effect and to test the crossed effect (i.e. across leaves) of leaf disc between different leaves. There was statistical evidence of a leaf mixed effect (LRT, p-value<0.0001), of a leaf disc location nested effect versus no leaf disc location effect (LRT, p-value<0.0001), and of a leaf disc location nested versus crossed effect (LRT, p-value<0.0001).

C. Relationship between Ct and dilution

We assumed that the relationship between the concentration c of the viroid RNA of the tested material and the Ct measurement is of the form $c = \kappa \times a^{-Ct} + \eta$, where a and κ are positive parameters, and η is a measure of residual error. The reciprocal relationship between Ct and c is then $Ct = (\log(\kappa) - \log(c))/\log(a) + \varepsilon$, where $\varepsilon = \log(1{+}\eta/(\kappa \times a^{-Ct}))/\log(a)$, or equivalently

$$Ct = K - A \times \log_{10}(c) + \varepsilon,$$

where $K = \log(\kappa)/\log(a)$ and $A = 1/\log_{10}(a)$.

When mixing an infected leaf i of concentration c_i with leaves $h_2, \dots, h_n$ of respective concentrations $c_{h_2}, \dots, c_{h_n} \ll c_i$, that are negligeable with respect to c_i, in a batch, the concentration of the batch is $\left(c_i + c_{h_2} + \dots + c_{h_n}\right)/n \approx c_i/n$.

A k –th repeated Ct measurements ($k = 1, \dots, j$) of the batch will be equal to $Ct_{batch,k} = K - A \times \log_{10}(c_i) + A \times \log_{10}(n) + \varepsilon_{batch,k}$, and a k –th repeated Ct measurement of the infected leaf Ct will be equal to $Ct_{i,j} = K - A \times \log_{10}(c_i) + \varepsilon_{i,k}$.

The relationship between the average of the batch technical replicate Ct measures and the average of the infected leaf technical replicates Ct measures will be:

$$\overline{Ct_{batch}} = \overline{Ct_i} + A \times \log_{10}(n) + (\overline{\varepsilon_{batch}} - \overline{\varepsilon_i}). \text{ (S1 Eqn 3)}$$

The variance of the residual term $\varepsilon = (\overline{\varepsilon_{batch}} - \overline{\varepsilon_i})$ is the sum of the variance due to the possible biological variations in batches of n leaves containing material from leaf i, and of the variance due to variations in repeated measures on the same biological material, this variance being inversely proportional to the number J of technical replicates: $\sigma_\varepsilon^2 = \sigma_{bio.rep.}^2 + \sigma_{tech.rep.}^2/\text{j}$.

D. Details of the computation of the risk function

Under the normality assumption of the residuals and the general model of the Ct measurements, an estimation of the probability that the batch Ct will be below the detection Ct when the batch contains l leaves with an expected Ct equal to Ct_{target} is $\tau(n, l) =$

$P\left(Ct_{target} + A(\times \log_{10}(n) - \log_{10}(l)) + \varepsilon < Ct_{detect}\right) =$

$\Phi\left(\frac{Ct_{detect} - Ct_{target} - A\times(log_{10}(n) - log_{10}(\mathrm{l}))}{\sigma_{\varepsilon}}\right)$ if L > 0, 0 otherwise.

The probability to select T infected trees when drawing n trees and sampling without replacement from a population containing m=$\lceil r \times N \rceil$ infected trees and N-m healthy trees is $h(N,\max(\lceil r \times N \rceil, 1), n_0 \times n_1, t)$ (where $\lceil . \rceil$ is the ceiling function and $h(N, m, n, t) = \left(\binom{N}{n}\right)^{-1} \binom{m}{t}\binom{N-m}{n-t}$ if $n + m - M \leq k \leq m$, 0 otherwise) is the probability to draw t infected trees without replacement in n draws from a population containing m infected trees and N-m non infected trees.

Let T be an integer between 0 and $n_0 \times n_1$. Let $(x_0, \dots, x_T)$ be a sequence of integers between 0 and n_1 such that $\sum_{i=0}^{T} i \times x_i = T$ and $\sum_{i=0}^{T} x_i = n_0$. If T is the number of infected trees selected in the sample and x_{i} be the number of batches that contain leaves from exactly I infected trees and $n_1 - I$ healthy trees, then conditionally on T, the probability to observe the distribution $(x_0, \dots, x_T)$ is exactly $\left(\frac{\prod_{i=0}^{T}\left(\frac{\mathrm{n}_1!}{i!(n_1-i)!}\right)^{x_{\mathrm{i}}}}{\frac{(n_0\times n_1)!}{T!(n_0\times n_1-T)!}}\right) \times \left(\frac{\mathrm{n}_0!}{\prod_{\mathrm{i}=0}\mathrm{x}_{\mathrm{i}}!}\right)$.

When a batch contains leaves from exactly i infected trees and $(n_1 - i)$ healthy trees, the probability to have l infected leaves in the batch and to have a negative result for the batch is $\binom{n_2\times i}{l}\beta^{n_2\times i-l}(1-\beta)^l\left(1-\tau(n_1\times n_2, l)\right)$. Overall, when a batch contains leaves from exactly i infected trees and $(n_1 - i)$ healthy trees, the probability to have a negative result for the batch is $\sum_{l=0}^{n_2\times i}\binom{n_2\times i}{l}\beta^{n_2\times i-l}(1-\beta)^l\left(1-\tau(n_1\times n_2, l)\right)$.

Overall, the risk to have a negative result for all the batches is lower than:

$$\sum_{t=0}^{n_0\times n_1}\left(\left(\left(\sum_{\substack{(x_0,\ldots,x_T)\in 0,\ldots,{n_1}^{T+1}\\ \sum_{i=0}^{T}x_i=n_0\\ \sum_{i=0}^{T}i\times x_i=T}}\left(\frac{\prod_{i=0}^{T}\left(\frac{n_1!}{i!\,(n_1-i)!}\right)^{x_i}}{\frac{(n_0\times n_1)!}{T!\,(n_0\times n_1-T)!}}\right)\times\left(\frac{n_0!}{\prod_{i=0}x_i\,!}\right)\right.\right.\right.$$

$$\times\left(\prod_{i=0}^{T}\left(\sum_{l=0}^{n_2\times i}\binom{n_2\times i}{l}\beta^{i\times n_2-l}(1-\beta)^l\left(1\right.\right.\right.$$

$$\left.\left.\left.\left.-\Phi\left(\frac{Ct_{detect}-Ct_{target}-A\times(log_{10}(n_1\times n_2)-log_{10}(l))}{\sigma_\varepsilon}\right)\right)\right)^{x_i}\right)\right)$$

$$\left.\times h(N,\max(\lceil r\times N\rceil,1),n_0\times n_1,t)\right) \qquad \text{(S1 Eqn 4).}$$

The computation of this probability is intractable for large values of $n_0 \times n_1$. And two approximations can be made. When the probability to get a positive result batch that contains material from a single infected leaf $\alpha_b = \Phi\left(\frac{Ct_{detect}-Ct_{target}-A\times(log_{10}(n_1\times n_2)-log_{10}(l))}{\sigma_\varepsilon}\right)$ tends to 1, then $1-\Phi\left(\frac{Ct_{detect}-Ct_{target}-A\times(log_{10}(n_1\times n_2)-log_{10}(l))}{\sigma_\varepsilon}\right)$ tends to 0 for L>0. So when α_b is close to 1, the overall risk of not detecting is close to $\sum_{t=0}^{n_0\times n_1}\left(\beta^t\times h(N,\max(\lceil r\times N\rceil,1),n_0\times n_1,t)\right)=E[exp(t\,log(\beta))]$, which is the moment generating function of the hypergeometric of parameters N (population size), $\max(\lceil r\times N\rceil,1)$ (success states), and

$n_0 \times n_1$ (sample size), applied to log(β), and also the probability that none of the n_0 batches contain material from an infected leaf. To account for the fact that there is a risk of not detecting the viroid in a batch that contains at least one infected leaf, another approximation of the intractable risk (5), valid when N, $r \times N$, and $n_0 \times n_1$are large is based on the approximation of the without replacement sampling by a with-replacement sampling. Under this approximation the risk is:

$$MajRisk(N, r, n_0, n_1, n_2, \beta)$$

$$= \left(\sum_{t=0}^{n_1} \left(\binom{n_1}{t} r^t (1 - r)^{n_1 - t} \sum_{l=0}^{t \times n_2} \left(\binom{n_2 \times t}{l} \beta^{t \times n_2 - l} (1 - \beta)^l \left(1 - \Phi \left(\frac{Ct_{detect} - Ct_{target} - A \times (log_{10}(n_1 \times n_2) - log_{10}(l))}{\sigma_\varepsilon} \right) \right) \right) \right) \right)^{n_0}.$$

This second approximation is preferred as it allows to take into account the risk of not detecting the viroid when present in a batch.

The interactive application allows 2500 independent simulations of an epidemic to be run for selected parameter sets (cf Table 1, main text). The application allows for three different selection methods, cluster sampling, simple random sampling and systematic sampling. Extensive simulations indicate that systematic sampling performs better than simple random sampling in reducing the risk of failure to detect the pathogen when it is present S1 Table 1). Systematic sampling is especially better than simple random sampling when the epidemic is concentrated in a small cluster, (reproduced in the simulations by setting the

number of sources of infections to 1) since simple random sampling may lead to samples being less regularly spaced throughout the target population[1]. The approximated risk for simple random sampling (used in Table 3 main text) can be treated as an upper bound for the risk in systematic sampling.

Sampling design	No. sources of infections	Risk		
		Approximation for simple random sampling	Expected relationship between approximated and simulated risk	Estimated via 2500 independent simulations. Estimated half width of confidence interval is following the estimate.
Simple random sampling	1	0.044	≈	0.044 ± 0.080
	10		≈	0.046 ± 0.082
Systematic sampling	1		>	0.042 ± 0.078
	10		>	0.040 ± 0.076

S1 Table 1: Risk of no detection and sampling design

The risk of no detection approximation and estimations for different sample schemes were obtained for the default parameters as listed in Table 1 (main text), for a field size of $N = 32000$, and a sample allocation of $n_0 = 35, n_1 = 53$, and $n_2 = 1$ all other parameters being the default parameters as in Table 1 main text.

E. Details on the optimization

Optimization involves a class of multistage stratified sampling designs associated with a pooling of samples process that does not disperse leaves from the same tree into different batches. The first stage corresponds to the selection of trees. The second stage corresponds to stratified selection of leaves, with constant allocation per stratum, and simple random

sampling within strata, where strata correspond to the division of the tree in octants or main branches.

To reduce the sample processing costs while obtaining the desirable confidence level for detection of the pathogen, leaves from multiple trees need to be tested in batches. The number of leaves per batch must not exceed the limit for which the Ct of a batch that contains a leaf below the target Ct will be higher than the detection Ct.

The estimated optimal allocation is defined as

$$(n_0^*, n_1^*, n_2^*) = \operatorname{argmin}\left\{\text{Cost}(n_0, n_1, n_2) \,|\, n_0, n_1, n_2 \in \mathbb{N}: \left\{\begin{array}{c} MajRisk(N, r, n_0, n_1, n_2, \beta) < 1 - \alpha, \\ \Phi\left((\widehat{\sigma}_\varepsilon)^{-1}\left(\text{Ct}_{\text{detect}} - \text{Ct}_{\text{target}} - \hat{A} \times \log_{10}(n_1 \times n_2)\right)\right) > \alpha_b \end{array}\right\}\right\}.$$

Note that $\Phi\left((\widehat{\sigma}_\varepsilon)^{-1}\left(\text{Ct}_{\text{detect}} - \text{Ct}_{\text{target}} - \hat{A} \times \log_{10}(n_1 \times n_2)\right)\right) > \alpha_b$ is equivalent to

$$n_1 \times n_2 < L = \exp\left(\hat{A}^{-1} \times \left(\text{Ct}_{\text{target}} - \text{Ct}_{\text{detect}} + \widehat{\sigma}_\varepsilon \times \Phi^{-1}(\alpha_b)\right) \times \log(10)\right) \quad \text{(S1 Eqn 5)}$$

In the case of non-unique optimal allocations, allocation with the smallest numbers of leaves per tree, or if equal with minimal number of trees per batch is returned by the algorithm.

F. Screenshots of the interactive web app

An interactive web-based app has been developed to illustrate the detection problem, to simulate the epidemic, sampling and detection processes (S1 Figure 3) and to allow computation of the optimal allocation (S1 Figure 4). The web app can be run via R through the ASBVdDetection package[2], or directly accessed[3].

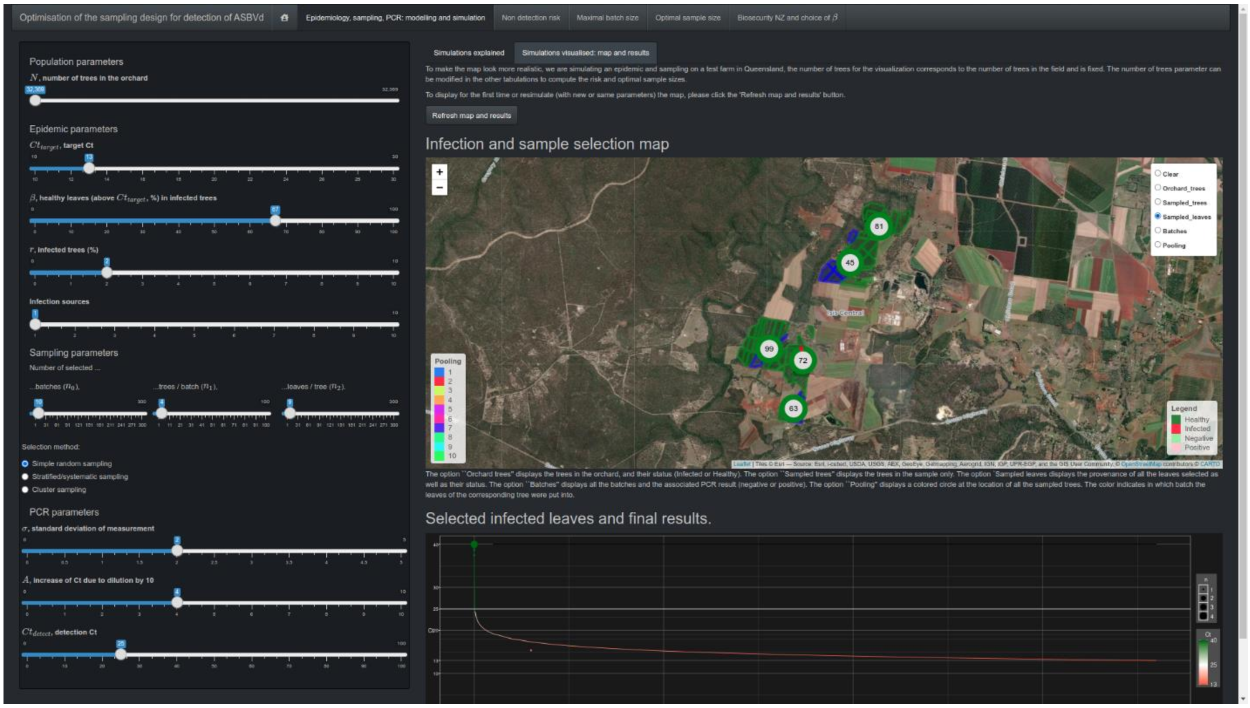

S1 Figure 3: Screenshot of the interactive application[3], second tabulation. This page allows the user to view the results of one simulation of an infection on an orchard, of the sampling and detection process. The process can be simulated a large number of times to check the validity of the formulae used for the risk (tabulation 3).

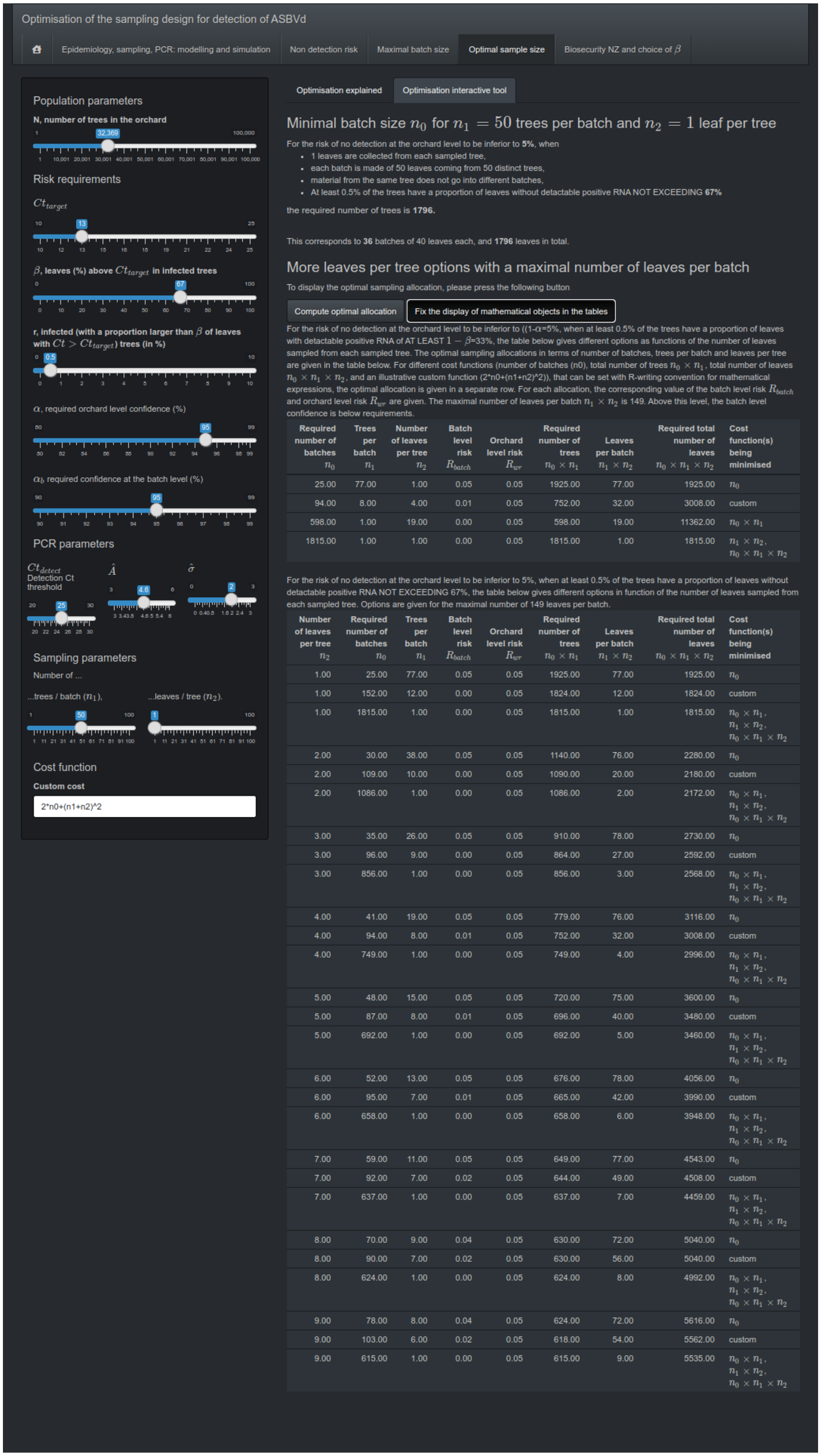

Minimal batch size n_0 for $n_1 = 50$ trees per batch and $n_2 = 1$ leaf per tree

For the risk of no detection at the orchard level to be inferior to **5%**, when

- 1 leaves are collected from each sampled tree,
- each batch is made of 50 leaves coming from 50 distinct trees,
- material from the same tree does not go into different batches,
- At least 0.5% of the trees have a proportion of leaves without detactable positive RNA NOT EXCEEDING **67%**

the required number of trees is **1796.**

This corresponds to **36** batches of 40 leaves each, and **1796** leaves in total.

More leaves per tree options with a maximal number of leaves per batch

To display the optimal sampling allocation, please press the following button

Compute optimal allocation | Fix the display of mathematical objects in the tables

For the risk of no detection at the orchard level to be inferior to ((1-α=5%, when at least 0.5% of the trees have a proportion of leaves with detactable positive RNA of AT LEAST $1-\beta$=33%, the table below gives different options as functions of the number of leaves sampled from each sampled tree. The optimal sampling allocations in terms of number of batches, trees per batch and leaves per tree are given in the table below. For different cost functions (number of batches (n0), total number of trees $n_0 \times n_1$, total number of leaves $n_0 \times n_1 \times n_2$, and an illustrative custom function (2*n0+(n1+n2)^2)), that can be set with R-writing convention for mathematical expressions, the optimal allocation is given in a separate row. For each allocation, the corresponding value of the batch level risk R_{batch} and orchard level risk R_{wr} are given. The maximal number of leaves per batch $n_1 \times n_2$ is 149. Above this level, the batch level confidence is below requirements.

Required number of batches n_0	Trees per batch n_1	Number of leaves per tree n_2	Batch level risk R_{batch}	Orchard level risk R_{wr}	Required number of trees $n_0 \times n_1$	Leaves per batch $n_1 \times n_2$	Required total number of leaves $n_0 \times n_1 \times n_2$	Cost function(s) being minimised
25.00	77.00	1.00	0.05	0.05	1925.00	77.00	1925.00	n_0
94.00	8.00	4.00	0.01	0.05	752.00	32.00	3008.00	custom
598.00	1.00	19.00	0.00	0.05	598.00	19.00	11362.00	$n_0 \times n_1$
1815.00	1.00	1.00	0.00	0.05	1815.00	1.00	1815.00	$n_1 \times n_2$, $n_0 \times n_1 \times n_2$

For the risk of no detection at the orchard level to be inferior to 5%, when at least 0.5% of the trees have a proportion of leaves without detactable positive RNA NOT EXCEEDING 67%, the table below gives different options in function of the number of leaves sampled from each sampled tree. Options are given for the maximal number of 149 leaves per batch.

Number of leaves per tree n_2	Required number of batches n_0	Trees per batch n_1	Batch level risk R_{batch}	Orchard level risk R_{wr}	Required number of trees $n_0 \times n_1$	Leaves per batch $n_1 \times n_2$	Required total number of leaves $n_0 \times n_1 \times n_2$	Cost function(s) being minimised
1.00	25.00	77.00	0.05	0.05	1925.00	77.00	1925.00	n_0
1.00	152.00	12.00	0.00	0.05	1824.00	12.00	1824.00	custom
1.00	1815.00	1.00	0.00	0.05	1815.00	1.00	1815.00	$n_0 \times n_1$, $n_1 \times n_2$, $n_0 \times n_1 \times n_2$
2.00	30.00	38.00	0.05	0.05	1140.00	76.00	2280.00	n_0
2.00	109.00	10.00	0.00	0.05	1090.00	20.00	2180.00	custom
2.00	1086.00	1.00	0.00	0.05	1086.00	2.00	2172.00	$n_0 \times n_1$, $n_1 \times n_2$, $n_0 \times n_1 \times n_2$
3.00	35.00	26.00	0.05	0.05	910.00	78.00	2730.00	n_0
3.00	96.00	9.00	0.00	0.05	864.00	27.00	2592.00	custom
3.00	856.00	1.00	0.00	0.05	856.00	3.00	2568.00	$n_0 \times n_1$, $n_1 \times n_2$, $n_0 \times n_1 \times n_2$
4.00	41.00	19.00	0.05	0.05	779.00	76.00	3116.00	n_0
4.00	94.00	8.00	0.01	0.05	752.00	32.00	3008.00	custom
4.00	749.00	1.00	0.00	0.05	749.00	4.00	2996.00	$n_0 \times n_1$, $n_1 \times n_2$, $n_0 \times n_1 \times n_2$
5.00	48.00	15.00	0.05	0.05	720.00	75.00	3600.00	n_0
5.00	87.00	8.00	0.01	0.05	696.00	40.00	3480.00	custom
5.00	692.00	1.00	0.00	0.05	692.00	5.00	3460.00	$n_0 \times n_1$, $n_1 \times n_2$, $n_0 \times n_1 \times n_2$
6.00	52.00	13.00	0.05	0.05	676.00	78.00	4056.00	n_0
6.00	95.00	7.00	0.01	0.05	665.00	42.00	3990.00	custom
6.00	658.00	1.00	0.00	0.05	658.00	6.00	3948.00	$n_0 \times n_1$, $n_1 \times n_2$, $n_0 \times n_1 \times n_2$
7.00	59.00	11.00	0.05	0.05	649.00	77.00	4543.00	n_0
7.00	92.00	7.00	0.02	0.05	644.00	49.00	4508.00	custom
7.00	637.00	1.00	0.00	0.05	637.00	7.00	4459.00	$n_0 \times n_1$, $n_1 \times n_2$, $n_0 \times n_1 \times n_2$
8.00	70.00	9.00	0.04	0.05	630.00	72.00	5040.00	n_0
8.00	90.00	7.00	0.02	0.05	630.00	56.00	5040.00	custom
8.00	624.00	1.00	0.00	0.05	624.00	8.00	4992.00	$n_0 \times n_1$, $n_1 \times n_2$, $n_0 \times n_1 \times n_2$
9.00	78.00	8.00	0.04	0.05	624.00	72.00	5616.00	n_0
9.00	103.00	6.00	0.02	0.05	618.00	54.00	5562.00	custom
9.00	615.00	1.00	0.00	0.05	615.00	9.00	5535.00	$n_0 \times n_1$, $n_1 \times n_2$, $n_0 \times n_1 \times n_2$

S1 Figure 4: screenshot of the interactive application[3], fifth tabulation. This page allows to compute the optimal allocation, for different cost functions (number of sample trees, number of sampled leaves per tree, number of sampled leaves as well as any custom function of n_0, n_1 and n_2.

References

1. De Jong PD. Sampling for detection: Leek rust as an example. International Journal of Pest Management. 1995;41: 31–35. doi:10.1080/09670879509371918
2. Bonnéry D B, Gilligan CA. ASBVdDetection R package. 2022. Available: https://gitlab.developers.cam.ac.uk/gilligan-epid/asbvddetect/asbvd-detect-r
3. Bonnéry D B, Gilligan C A. ASBVdSampling interactive application. 2022. Available: https://danielbonnery.shinyapps.io/ASBVd_Sampling
