# Supplementary figures and images for: Rational design of a survey protocol for avocado sunblotch viroid in commercial orchards to demonstrate pest freedom
Source: PLoS One. 2023 Apr 11;18(4):e0277725. doi: 10.1371/journal.pone.0277725 (PMC10089318; doi:10.1371/journal.pone.0277725)

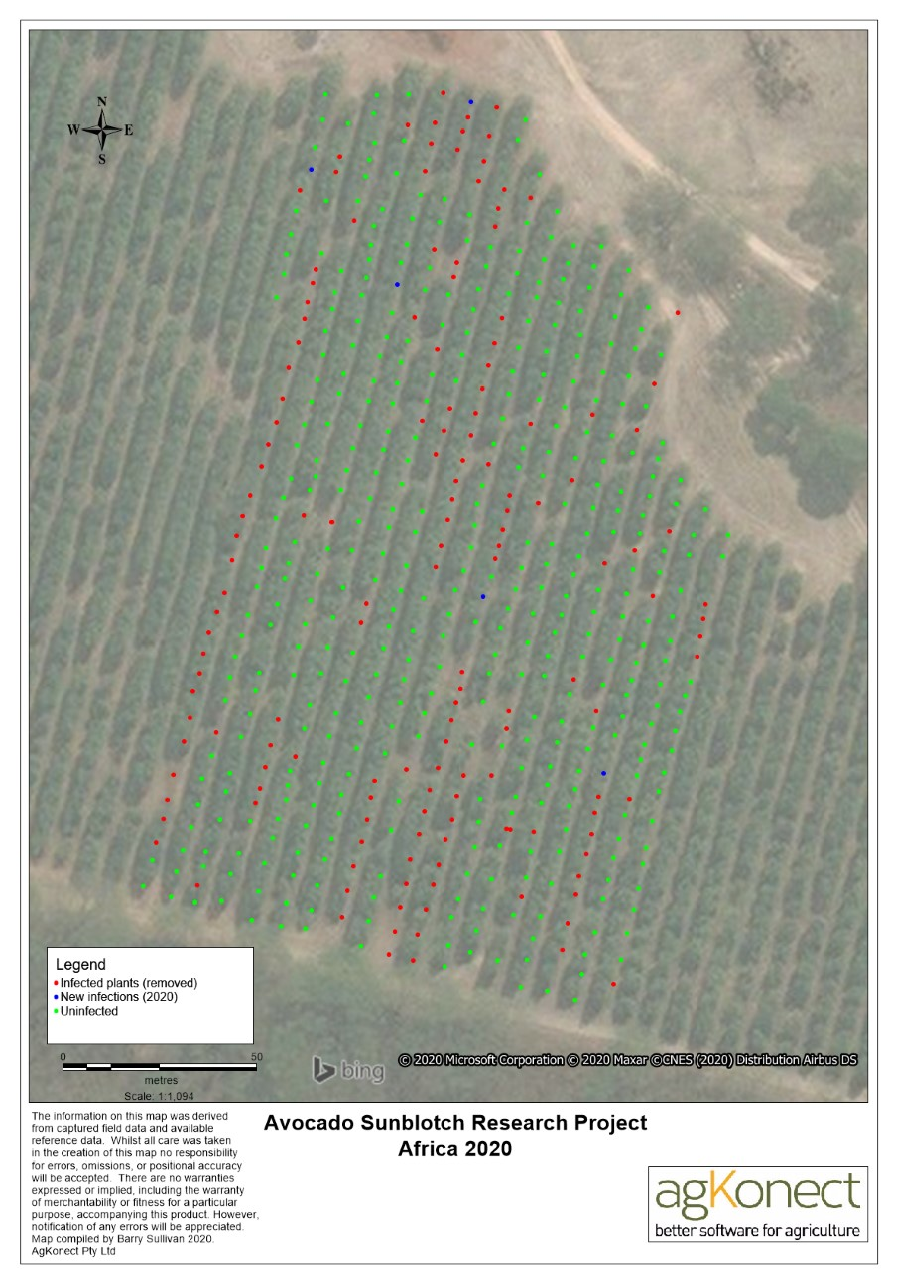

Supplement: S2 File — The archive file asbvd-detect-r.tar.gz is an R package that contains the source code and data used for all the statistical analysis, as well as the source code for the interactive tool [22]. It is a copy of the source code available at [26]. (GZ) [file pone.0277725.s002.gz › asbvd-detect-r-main/inst/extdata/SA_EJ_Nelspruit.tif]
