# Supplementary material for: Rational design of a survey protocol for avocado sunblotch viroid in commercial orchards to demonstrate pest freedom
Source: PLoS One. 2023 Apr 11;18(4):e0277725. doi: 10.1371/journal.pone.0277725 (PMC10089318; doi:10.1371/journal.pone.0277725)
Supplement: Attachment — Submitted filename: PONE-D-22-30012 Review comments.pdf [file pone.0277725.s003.pdf]

Manuscript: PONE-D-22-30012

Title: Rational design of a sampling protocol for demonstrating area freedom from a subcellular plant pathogen in commercial orchards

Authors: Bonnéry et al.

Overview: This manuscript describes a protocol for field sampling and detection of ASBVd that can be used as a regulatory procedure which, if samples from a field or region are negative for ASBVd, can support export of avocado to a country that does not accept import of avocado from a region or field that has ASBVd. I believe the title is too broad (e.g. subcellular plant pathogen) and should be specific for avocado and ASBVd. Many other viruses and viroids can be considered subcellular and the filter disk capture of the nucleic acid template may not be considered robust enough as part of a regulatory detection platform. Moreover, the molecular description of ASBVd is not adequately described to provide background needed to evaluate template preparation and RT-qPCR methods used. I do not like the term “negotiable” regarding an export protocol. Rather, it is based on solid data or a series of criteria that are “mutually” accepted.

Specific comments:

Abstract

Pg1. Suggest replacing “negotiable” with “critical…”

Pg 2. Suggest replacing “flexible” with “scalable.”

Introduction

Pg 3. Seed transmission rates of 86-100%? What is the citation? Wallace and Drake (1953) reported rate of seed-transmission from symptomatic ASBVd-infected trees of 5%. Also, what are examples symptomless carrier strains?

Pg. 4. Provide better molecular description along with the biological characters of ASBVd.

Pg 5. Batch-testing is better referred to as the number of individual plants or trees that can be combined as one sample rather than simply the number of leaves.

Pg 6. I did not want to read Ref 16 but I think a citation from an plant disease journal id preferable to a paper in a veterinary journal.

M&M

Pg. 8. In Austria PCR ran for 40 cycles, whereas in So. Africa, 35 cycles. In Table 2 the “target Ct is 15. It is unclear what constitutes a “positive” sample. By batching samples, the positive threshold needs to be well defined along with a measure (quality assessment) of the RNA extraction method such as an RNA host target of NADPH. How many PCR plate wells were included as duplicated samples to account for pipet errors/variations. This is a critical factor as the Ct is used for your mathematical (modeling) estimates?

Pg. 9. Clarify “Potential patterning (privileged area)”.

Pg10. Expt 3. It seems that mixing an extract from one ASBVd-positive leaf disk with 199 negative leaf disks is an extreme stretch of target PCR limits due to PCR inhibitors and random priming. Again, what is the criteria of a PCR-positive sample?

Pg.10. Eq. 1. I understand Ct_{batch} but not $Ct_{individual}$ for large number of samples. Clarify S1 Section C did **not help make this clear.**

Results

Pg.13. I was unable to open the "app".

Pg.12-20. My mathematical expertise is limited so won't comment on this section.

Discussion

Pg 20-23. As mentioned above, my mathematical expertise is limited so I can't fairly evaluate the results. However, it seems the hierarchical bulk sampling portion would benefit by using a real-life field rather than only present results from modeling. For example, how many samples from trees can you safely batch to be able to discern a ASBVd-free orchard from one that has 1, 5, 10% infection rate? Seems this protocol has a pretty good change of missing an occasional ASBVd-infected tree. Moreover, I am not convinced of root-to-root transmission of ASBVd. In the case of other viroids of which I am familiar, mechanical transmission with pruning or cultivating equipment can be an efficient method of viroid spread from an inoculum source. I could not tell if this was accounted for in the dynamics of the model presented. Methods used to establishing a pest-free status for trade, indeed, needs a robust protocol and I am not able to judge how effective the methods described achieves this.
