# Supplementary material for: Rational design of a survey protocol for avocado sunblotch viroid in commercial orchards to demonstrate pest freedom
Source: PLoS One. 2023 Apr 11;18(4):e0277725. doi: 10.1371/journal.pone.0277725 (PMC10089318; doi:10.1371/journal.pone.0277725)
Supplement: Attachment — Submitted filename: Rebuttal letter (2_.docx [file pone.0277725.s004.docx]

# Rational design of a survey protocol for avocado sunblotch viroid in commercial orchards to demonstrate pest freedom

# Authors’ responses to Reviewers and Editor

To the editor: Thank you for the prompt handling of our manuscript and for the helpful editorial comments and advice. Please find below our detailed responses in the inset boxes to the questions and comments from the referees and yourself. We also made minor edits that were not asked by the referees or you and we list these edits at the end of this document.

**Editor**: *Thank you for submitting your manuscript to PLOS ONE. After careful consideration, we feel that it has merit but does not fully meet PLOS ONE’s publication criteria as it currently stands. Therefore, we invite you to submit a revised version of the manuscript that addresses the points raised during the review process.*
*This is a research protocol paper. I expect widely acceptable details on method(s). It seems to me you had it but needs to clarify some details as one of the reviewers showed. I think your title is beyond what you suggest in general although the method might be used for other crops but needs to be verified as a protocol. I hope you will be able to make clearer statements.*

**Response:** Thank you for your constructive suggestions.

**Action:** We have amended the title and provided some additional explanations to improve the clarity and scope of the paper. The title now reads: Rational design of a survey protocol for avocado sunblotch viroid in commercial orchards to demonstrate pest freedom.

**Editor:** *Please submit your revised manuscript by Feb 25* *2023 11:59PM. If you will need more time than this to complete your revisions, please reply to this message or contact the journal office at* [*plosone@plos.org*](mailto:plosone@plos.org)*. When you're ready to submit your revision, log on to* [*https://www.editorialmanager.com/pone/*](https://eur03.safelinks.protection.outlook.com/?url=https%3A%2F%2Fwww.editorialmanager.com%2Fpone%2F&data=05%7C01%7Cdbb31%40universityofcambridgecloud.onmicrosoft.com%7C0f4ccd874e974af3872308daf3bf6860%7C49a50445bdfa4b79ade3547b4f3986e9%7C1%7C0%7C638090301184416315%7CUnknown%7CTWFpbGZsb3d8eyJWIjoiMC4wLjAwMDAiLCJQIjoiV2luMzIiLCJBTiI6Ik1haWwiLCJXVCI6Mn0%3D%7C3000%7C%7C%7C&sdata=vajOn48QSMAZCn6Gik46IEQXTg2jTERB1BADT60wrV0%3D&reserved=0) *and select the 'Submissions Needing Revision' folder to locate your manuscript file.*

*Please include the following items when submitting your revised manuscript:*

- *A rebuttal letter that responds to each point raised by the academic editor and reviewer(s). You should upload this letter as a separate file labeled 'Response to Reviewers'.*
- *A marked-up copy of your manuscript that highlights changes made to the original version. You should upload this as a separate file labeled 'Revised Manuscript with Track Changes'.*
- *An unmarked version of your revised paper without tracked changes. You should upload this as a separate file labeled 'Manuscript'.*

**Action**: Done.

**Editor:** *If you would like to make changes to your financial disclosure, please include your updated statement in your cover letter.*

**Response:** No changes are required to our financial disclosure.

**Editor:** *Guidelines for resubmitting your figure files are available below the reviewer comments at the end of this letter.*

**Response:** We thank you for this reminder. We have followed the guidelines.

**Editor:** *If applicable, we recommend that you deposit your laboratory protocols in* [*protocols.io*](https://eur03.safelinks.protection.outlook.com/?url=http%3A%2F%2Fprotocols.io%2F&data=05%7C01%7Cdbb31%40universityofcambridgecloud.onmicrosoft.com%7C0f4ccd874e974af3872308daf3bf6860%7C49a50445bdfa4b79ade3547b4f3986e9%7C1%7C0%7C638090301184416315%7CUnknown%7CTWFpbGZsb3d8eyJWIjoiMC4wLjAwMDAiLCJQIjoiV2luMzIiLCJBTiI6Ik1haWwiLCJXVCI6Mn0%3D%7C3000%7C%7C%7C&sdata=jdqHlfMr4jaYOB4bvivfpVf0yB9Z68DwQrxPxkgnRT4%3D&reserved=0) *to enhance the reproducibility of your results. Protocols.io assigns your protocol its own identifier (DOI) so that it can be cited independently in the future. For instructions see:* [*https://journals.plos.org/plosone/s/submission-guidelines#loc-laboratory-protocols*](https://eur03.safelinks.protection.outlook.com/?url=https%3A%2F%2Fjournals.plos.org%2Fplosone%2Fs%2Fsubmission-guidelines%23loc-laboratory-protocols&data=05%7C01%7Cdbb31%40universityofcambridgecloud.onmicrosoft.com%7C0f4ccd874e974af3872308daf3bf6860%7C49a50445bdfa4b79ade3547b4f3986e9%7C1%7C0%7C638090301184416315%7CUnknown%7CTWFpbGZsb3d8eyJWIjoiMC4wLjAwMDAiLCJQIjoiV2luMzIiLCJBTiI6Ik1haWwiLCJXVCI6Mn0%3D%7C3000%7C%7C%7C&sdata=7GSXTa5FZStMfTSoDJjPx1Qn3OR1ilynOQ44U5wZvDI%3D&reserved=0)*. Additionally, PLOS ONE offers an option for publishing peer-reviewed Lab Protocol articles, which describe protocols hosted on* [*protocols.io*](https://eur03.safelinks.protection.outlook.com/?url=http%3A%2F%2Fprotocols.io%2F&data=05%7C01%7Cdbb31%40universityofcambridgecloud.onmicrosoft.com%7C0f4ccd874e974af3872308daf3bf6860%7C49a50445bdfa4b79ade3547b4f3986e9%7C1%7C0%7C638090301184572534%7CUnknown%7CTWFpbGZsb3d8eyJWIjoiMC4wLjAwMDAiLCJQIjoiV2luMzIiLCJBTiI6Ik1haWwiLCJXVCI6Mn0%3D%7C3000%7C%7C%7C&sdata=r4ywsuoXqkSTBjrV5Ny6JxotajyKKewNe2vX%2BP6M5K4%3D&reserved=0)*. Read more information on sharing protocols at* [*https://plos.org/protocols?utm_medium=editorial-email&utm_source=authorletters&utm_campaign=protocols*](https://eur03.safelinks.protection.outlook.com/?url=https%3A%2F%2Fplos.org%2Fprotocols%3Futm_medium%3Deditorial-email%26utm_source%3Dauthorletters%26utm_campaign%3Dprotocols&data=05%7C01%7Cdbb31%40universityofcambridgecloud.onmicrosoft.com%7C0f4ccd874e974af3872308daf3bf6860%7C49a50445bdfa4b79ade3547b4f3986e9%7C1%7C0%7C638090301184572534%7CUnknown%7CTWFpbGZsb3d8eyJWIjoiMC4wLjAwMDAiLCJQIjoiV2luMzIiLCJBTiI6Ik1haWwiLCJXVCI6Mn0%3D%7C3000%7C%7C%7C&sdata=4d8orcWF9oSsyENsQHsH6DBJ1H4DH6Witm9AUtUcwf4%3D&reserved=0)*.*

**Response:** We use standard protocols for detection and quantification of the viroid that is described and supported with appropriate references in the text and so we do not consider it necessary to deposit this protocol in protocols.io at this time.

**Action:** We do, however, provide a link to a publicly available app (Reference [22] of the paper) to accompany the paper that allows users to compute sampling designs for pest freedom.

**Editor:** *Journal Requirements:*

*When submitting your revision, we need you to address these additional requirements.*

1. *Please ensure that your manuscript meets PLOS ONE’s style requirements, including those for file naming. The PLOS ONE style templates can be found at*

[*https://journals.plos.org/plosone/s/file?id=wjVg/PLOSOne_formatting_sample_main_body.pdf*](https://eur03.safelinks.protection.outlook.com/?url=https%3A%2F%2Fjournals.plos.org%2Fplosone%2Fs%2Ffile%3Fid%3DwjVg%2FPLOSOne_formatting_sample_main_body.pdf&data=05%7C01%7Cdbb31%40universityofcambridgecloud.onmicrosoft.com%7C0f4ccd874e974af3872308daf3bf6860%7C49a50445bdfa4b79ade3547b4f3986e9%7C1%7C0%7C638090301184572534%7CUnknown%7CTWFpbGZsb3d8eyJWIjoiMC4wLjAwMDAiLCJQIjoiV2luMzIiLCJBTiI6Ik1haWwiLCJXVCI6Mn0%3D%7C3000%7C%7C%7C&sdata=Yd%2BAf5fjGsoTsWNqi%2FRO2wspEEmJjxS5fvohIJjjwMY%3D&reserved=0) *and*

[*https://journals.plos.org/plosone/s/file?id=ba62/PLOSOne_formatting_sample_title_authors_affiliations.pdf*](https://eur03.safelinks.protection.outlook.com/?url=https%3A%2F%2Fjournals.plos.org%2Fplosone%2Fs%2Ffile%3Fid%3Dba62%2FPLOSOne_formatting_sample_title_authors_affiliations.pdf&data=05%7C01%7Cdbb31%40universityofcambridgecloud.onmicrosoft.com%7C0f4ccd874e974af3872308daf3bf6860%7C49a50445bdfa4b79ade3547b4f3986e9%7C1%7C0%7C638090301184572534%7CUnknown%7CTWFpbGZsb3d8eyJWIjoiMC4wLjAwMDAiLCJQIjoiV2luMzIiLCJBTiI6Ik1haWwiLCJXVCI6Mn0%3D%7C3000%7C%7C%7C&sdata=%2BcpB%2BH2VvzTkPjuQVnSn%2FKuRqguQfrSyCOPPOVLGxd4%3D&reserved=0)

**Action:** Done and checked.

**Editor:** *2. Thank you for stating the following in the Acknowledgments Section of your manuscript:*

*“This project has been funded by Hort Innovation, using the avocado research and*

*development levy and contributions from the Australian Government under Project*

*AV18007 – Avocado sunblotch viroid survey. Hort Innovation is the grower owned, not-forprofit research and development corporation for Australian horticulture.” We note that you have provided additional information within the Acknowledgements Section that is not currently declared in your Funding Statement. Please note that funding information should not appear in the Acknowledgments section or other areas of your manuscript. We will only publish funding information present in the Funding Statement section of the online submission form.*

*Please remove any funding-related text from the manuscript and let us know how you would like to update your Funding Statement. Currently, your Funding Statement reads as follows:*

*“This project has been funded by Hort Innovation (*[*https://www.horticulture.com.au/*](https://eur03.safelinks.protection.outlook.com/?url=https%3A%2F%2Fwww.horticulture.com.au%2F&data=05%7C01%7Cdbb31%40universityofcambridgecloud.onmicrosoft.com%7C0f4ccd874e974af3872308daf3bf6860%7C49a50445bdfa4b79ade3547b4f3986e9%7C1%7C0%7C638090301184572534%7CUnknown%7CTWFpbGZsb3d8eyJWIjoiMC4wLjAwMDAiLCJQIjoiV2luMzIiLCJBTiI6Ik1haWwiLCJXVCI6Mn0%3D%7C3000%7C%7C%7C&sdata=vLlIiRnELJs2UYm53s6TgIf3O0nt2IcZIVuobs6D0QU%3D&reserved=0)*), using the avocado research and development levy and contributions from the Australian Government under Project AV18007 – Avocado sunblotch viroid survey. Hort Innovation is the grower owned, not-for-profit research and development corporation for Australian horticulture. The grant was received by A. G. The funders had no role in study design, data collection and analysis, decision to publish, or preparation of the manuscript.”*

*Please include your amended statements within your cover letter; we will change the online submission form on your behalf.*

Action: 1) Noted: we removed the following sentence from the Acknowledgement section of the manuscript.

This project has been funded by Hort Innovation, using the avocado research and development levy and contributions from the Australian Government under Project AV18007 – Avocado sunblotch viroid survey. Hort Innovation is the grower owned, not-for-profit research and development corporation for Australian horticulture.

2) We do not wish to amend the following funding statement, that we put in the cover letter and in the submission form.

This project has been funded by Hort Innovation ([https://www.horticulture.com.au/](https://eur03.safelinks.protection.outlook.com/?url=https%3A%2F%2Fwww.horticulture.com.au%2F&data=05%7C01%7Cdbb31%40universityofcambridgecloud.onmicrosoft.com%7C0f4ccd874e974af3872308daf3bf6860%7C49a50445bdfa4b79ade3547b4f3986e9%7C1%7C0%7C638090301184572534%7CUnknown%7CTWFpbGZsb3d8eyJWIjoiMC4wLjAwMDAiLCJQIjoiV2luMzIiLCJBTiI6Ik1haWwiLCJXVCI6Mn0%3D%7C3000%7C%7C%7C&sdata=vLlIiRnELJs2UYm53s6TgIf3O0nt2IcZIVuobs6D0QU%3D&reserved=0)), using the avocado research and development levy and contributions from the Australian Government under Project AV18007 – Avocado sunblotch viroid survey. Hort Innovation is the grower owned, not-for-profit research and development corporation for Australian horticulture. The grant was received by A. G. The funders had no role in study design, data collection and analysis, decision to publish, or preparation of the manuscript.

**Editor:** *3. Please include your full ethics statement in the ‘Methods’ section of your manuscript file. In your statement, please include the full name of the IRB or ethics committee who approved or waived your study, as well as whether or not you obtained informed written or verbal consent. If consent was waived for your study, please include this information in your statement as well.*

Action: Noted: a subsection of the Methods Section has been duly added and now reads:

“**Ethics Statement**

Researchers undertaking field assessments in Australia and South Africa obtained verbal consent from landowners and farm managers to access the avocado orchards and collect leaf samples for the purpose of these investigations. With property owners consent the Biosecurity Queensland was notified of the presence of the infected trees in Australia and authorisation provided to retain the trees for the experimental purposes described in this paper.”

**Editor:** *4. Please include captions for your Supporting Information files at the end of your manuscript, and update any in-text citations to match accordingly. Please see our Supporting Information guidelines for more information:* [*http://journals.plos.org/plosone/s/supporting-information*](https://eur03.safelinks.protection.outlook.com/?url=http%3A%2F%2Fjournals.plos.org%2Fplosone%2Fs%2Fsupporting-information&data=05%7C01%7Cdbb31%40universityofcambridgecloud.onmicrosoft.com%7C0f4ccd874e974af3872308daf3bf6860%7C49a50445bdfa4b79ade3547b4f3986e9%7C1%7C0%7C638090301184572534%7CUnknown%7CTWFpbGZsb3d8eyJWIjoiMC4wLjAwMDAiLCJQIjoiV2luMzIiLCJBTiI6Ik1haWwiLCJXVCI6Mn0%3D%7C3000%7C%7C%7C&sdata=mCFDFI8kskiUbvaL449n6nU6x09KRKSFmjEM2nd9ebk%3D&reserved=0)*.*

**Action:** Noted: the captions for our Supporting Information files at the end of our manuscript are now:

S1 Additional figures, mathematical formulas and presentation of the interactive computation tool

S2 R package. ASBVdDetection

The archive file asbvd-detect-r.tar.gz is an R package that contains the source code and data used for all the statistical analysis, as well as the source code for the interactive tool [22]. It is a copy of the source code available at [26].

**Editor:** *Additional Editor Comments:*

*Two reviewers have suggested different decisions deeply. I would like to have your responds on reviewers’ comments, especially from one who has rejected. I* *definitely agree with editor on too broad title you have.* *I think you need to explain methodic problems carefully.*

**Response:** We thank you and the Reviewers for the comments.

**Action:** We have changed the title to make it more specific. We have responded to each of the concerns raised by Reviewer #2, below.

**Editor:** *Reviewers’ comments:*

*Reviewer’s Responses to Questions*

*Comments to the Author*

*1. Is the manuscript technically sound, and do the data support the conclusions?*

*The manuscript must describe a technically sound piece of scientific research with data that supports the conclusions. Experiments must have been conducted rigorously, with appropriate controls, replication, and sample sizes. The conclusions must be drawn appropriately based on the data presented.*

*Reviewer #1: Yes*

*Reviewer #2: Partly*

**Response:** We note that Reviewer #1 considers the manuscript to be technically sound and we address the specific concerns raised by Reviewer #2’s below.

**Editor:** *2. Has the statistical analysis been performed appropriately and rigorously?*

*Reviewer #1: Yes*

*Reviewer #2: I Don’t Know*

**Response:** No action given that reviewer #1 is content.

**Editor:** *3. Have the authors made all data underlying the findings in their manuscript fully available?*
*The* [*PLOS Data policy*](https://eur03.safelinks.protection.outlook.com/?url=http%3A%2F%2Fwww.plosone.org%2Fstatic%2Fpolicies.action%23sharing&data=05%7C01%7Cdbb31%40universityofcambridgecloud.onmicrosoft.com%7C0f4ccd874e974af3872308daf3bf6860%7C49a50445bdfa4b79ade3547b4f3986e9%7C1%7C0%7C638090301184572534%7CUnknown%7CTWFpbGZsb3d8eyJWIjoiMC4wLjAwMDAiLCJQIjoiV2luMzIiLCJBTiI6Ik1haWwiLCJXVCI6Mn0%3D%7C3000%7C%7C%7C&sdata=rlpJDebp6HTtTBjaI6OZ0FjRU%2FLAs9g%2FBqZxdcq0edw%3D&reserved=0) *requires authors to make all data underlying the findings described in their manuscript fully available without restriction, with rare exception (please refer to the Data Availability Statement in the manuscript PDF file). The data should be provided as part of the manuscript or its supporting* *information, or deposited to a public repository. For example, in addition to summary statistics, the data points behind means, medians and variance measures should be available. If there are restrictions on publicly sharing data—**e.g. participant privacy or use of data from a third party—those must be specified.*

*Reviewer #1: Yes*

*Reviewer #2: No*

**Response:** We have included all the data in the R data package (reference 26). The Supporting Information (S2) also contains the programs used to produce the different outputs of the statistical analysis and describes how to use the programs in the GitLab pages (reference 27). We are concerned that Reviewer 2 was unable to open the app (available on a website at <https://danielbonnery.shinyapps.io/ASBVd_Sampling/>) which formed a support for our work, and therefore was unable to access the full submission – we can only suggest this was due to a temporary or internal technical issue.

We note reviewer #1 had full access and we have checked with co-authors and colleagues in Europe, Australia and South Africa that access to this app is accessible and can confirm that this is the case.

**Editor:** *4. Is the manuscript presented in an intelligible fashion and written in standard English?*
*PLOS ONE does not copyedit accepted manuscripts, so the language in submitted articles must be clear, correct, and unambiguous. Any typographical or grammatical errors should be corrected at revision, so please note any specific errors here.*

*Reviewer #1: Yes*

*Reviewer #2: No*

**Response:** We note that Reviewer #1 is content and Reviewer #2 does not indicate explicit concerns here.

**Action:** We have carefully reviewed the manuscript (making a number of typographical and grammatical changes, marked under tracked changes) and are content with the language.

***Reviewer 1***: *5. Review Comments to the* *Author[…]*

*Dear Editor,The study presents the results of original research and its results reported have not been published elsewhere. Experiments, statistics, and other analyses are performed to a high technical standard and are described in sufficient detail. Conclusions are presented in an appropriate fashion and are supported by the data. The article is presented in an intelligible fashion and is written in good English. The research meets all applicable standards for the ethics of experimentation and research integrity. The article adheres to appropriate reporting guidelines and community standards for data availability. However, I suggest some minor revisions. Introduction of the paper is too* *long and readers can get bored/tired. Please, shorten it if applicable.* *And, my decision is to accept the article after the minor revisions.*

**Response:** We thank Reviewer #1 for these positive comments on the language and presentation of the manuscript and confirmation that it meets the journal’s requirements on ethics and research integrity, including standards for data availability.

**Action:** We have carefully reviewed the introduction and we feel that it is necessary to provide the reader with context about the crop, free trade agreements with respect to pest free status and then to introduce the current relevant knowledge of avocado sunblotch and methods for detection. Accordingly, we wish to retain the current form of the Introduction.

***Reviewer 2:*** *5. Review Comments to the Author […]: This manuscript describes a protocol for field sampling and detection of* *ASBVd that can be used as a regulatory procedure which, if samples from a field or region are negative for ASBVd, can support export of avocado to a country that does not accept import of avocado from a region or field that has ASBVd. I believe the title is too broad (**e.g. subcellular plant pathogen) and should be specific for avocado and ASBVd. Many other viruses and viroids can be considered* *subcellular and the filter disk capture of the nucleic acid template may not be considered robust enough as part of a regulatory detection platform.*

**Response:** We thank the reviewer for the general appraisal.

**Action:** We agree that the title is too broad and it has been changes to: Rational design of a survey protocol for avocado sunblotch viroid in commercial orchards to demonstrate pest freedom.

***Reviewer 2:*** *Moreover, the molecular description of ASBVd is not adequately described to provide background needed to evaluate template preparation and RT-qPCR methods used. I do not like the term “negotiable” regarding an export protocol. Rather, it is based on solid data or a series of criteria that are “mutually” accepted. This and specific comments included in the attachment.*

**Response:** The novelty of our work is in the design, development and implementation of a statistical protocol to optimize sampling for pest freedom, using a PCR protocol and critical values for prevalence and confidence intervals that are agreed by the regulatory authorities. We have introduced the molecular (and symptomatic) descriptions citing supporting references of authors who have recently reviewed the pathogen in depth (e.g., Khun et al 2017; Saucedo Carabez et al 2019). Sufficient details are given to allow reproduction of the experiments described in the paper but we consider detailed molecular description of ASBVd to be unnecessary and beyond the scope of the appear.

***Editor:*** *While revising your submission, please upload your figure files to the Preflight Analysis and Conversion Engine (PACE) digital diagnostic tool,* [*https://pacev2.apexcovantage.com/*](https://eur03.safelinks.protection.outlook.com/?url=https%3A%2F%2Fpacev2.apexcovantage.com%2F&data=05%7C01%7Cdbb31%40universityofcambridgecloud.onmicrosoft.com%7C0f4ccd874e974af3872308daf3bf6860%7C49a50445bdfa4b79ade3547b4f3986e9%7C1%7C0%7C638090301184572534%7CUnknown%7CTWFpbGZsb3d8eyJWIjoiMC4wLjAwMDAiLCJQIjoiV2luMzIiLCJBTiI6Ik1haWwiLCJXVCI6Mn0%3D%7C3000%7C%7C%7C&sdata=Qvh6coAf7H9eFgiCiVDQ23AVBxGclGvb93c7nWZcGs4%3D&reserved=0)*. PACE helps ensure that figures meet PLOS requirements. To use PACE, you must first register as a user. Registration is free. Then, login and navigate to the UPLOAD tab, where you will find detailed instructions on how to use the tool. If you encounter any issues or have any questions when using PACE, please email PLOS at* [*figures@plos.org*](mailto:figures@plos.org)*. Please note that Supporting Information files do not need this step.*

**Response:** We did not modify the figures in our second submission and had successfully used the PACE diagnostic tool to check all the figures that went into the main manuscript.

***Editor:*** *If reviewer comments were submitted as an attachment file, they will be attached to this email and accessible via the submission site. Please log into your account, locate the manuscript record, and check for the action link "View Attachments". If this link does not appear, there are no attachment files.*

**Response:** Only reviewer 2 uploaded an attachment, our answers to points raised by Reviewer 2 in the attachments are below.

# Response to points raised in the attachments

***Reviewer 2:*** *Overview: This manuscript describes a protocol for field sampling and detection of ASBVd that can be used as a regulatory procedure which, if samples from a field or region are negative for ASBVd, can support export of avocado to a country that does not accept import of avocado from a region or field that has ASBVd. I believe the title is too broad (**e.g. subcellular plant pathogen) and should be specific for avocado and ASBVd. Many other viruses and viroids can be considered* *subcellular and the filter disk capture of the nucleic acid template may not be considered robust enough as part of a regulatory detection platform. Moreover, the molecular description of ASBVd is not adequately described to provide background needed to evaluate template preparation and RT-qPCR methods used. I do not like the term “negotiable” regarding an export protocol. Rather, it is based on solid data or a series of criteria that are “mutually” accepted.*

**Response:** We agree with the reviewer that the original title was too broad and the title has been changed to be more specific. We do not feel it is appropriate, however, to describe more details on the molecular characteristics of ASBVd as these details are not relevant to the paper. The filter paper method of viroid RNA extraction is highly sensitive and comparable with other RNA extraction methods, as described by the cited paper, Pretorius and Geering (2022). The aim of the experiments described in this paper was to show how samples could be bulked to economise on testing, and to provide a statistical method to decide how many samples should be combined to form a batch.

**Action:** Title changed to make it more specific.

***Reviewer 2:*** *Specific comments:*

*Abstract*

*Pg1. Suggest replacing “negotiable” with “critical…”*

**Action:** We see now that the sentence was ambiguous. We have removed ‘negotiable’ entirely and the sentence now reads more clearly.

***Reviewer 2:*** *Pg 2. Suggest replacing “flexible” with “scalable.”*

**Action:** Thank you for this preferable alternative: we have replaced flexible with scalable.

***Reviewer 2:*** *Introduction*

*Pg 3. Seed transmission rates of 86-100%? What is the citation? Wallace and Drake (1953) reported rate of seed-transmission from symptomatic ASBVd-infected trees of 5%. Also, what are examples symptomless carrier strains?*

**Action:** Apologies: an incorrect paper was cited, and the reference is now corrected to: “Wallace and Drake (1962) record transmission rates of 86-100% in seed from an asymptomatically infected tree (see Tables 1 and 2 in this paper).”

***Reviewer 2:*** *Pg. 4. Provide better molecular description along with the biological characters of ASBVd.*

**Response:** We have introduced the molecular (and symptomatic) descriptions, citing supporting references of authors who have recently reviewed the pathogen in depth (e.g. Khun et al 2017; Saucedo Carabez et al 2019), which we believe is an appropriate level of detail for the paper for reasons given above we do not consider it necessary to describe more details on the molecular characteristics of ASBVd since the paper is about the design of a survey protocol to meet regulatory requirements to demonstrate pest-freedom.

***Reviewer 2:*** *Pg 5. Batch-testing is better referred to as the number of individual plants or trees that can be combined as one sample rather than simply the number of leaves.*

**Action:** We have clarified the terminology in the paper to make it clear that batch-testing refers to aggregations of multiple leaf samples from within and between trees.

***Reviewer 2:*** *Pg 6. I did not want to read Ref* *16 but I think a citation from* *an plant disease journal id preferable to a paper in a veterinary journal.*

**Response:** Ref 16 refers to: Cameron AR, Baldock FC. “Two-stage sampling in surveys to substantiate freedom from disease. Preventive Veterinary Medicine.” 1998;34: 19–30. doi:10.1016/S0167-5877(97)00073-1. Just to clarify our reasoning for selecting this reference, our paper is about sampling strategies for detection of a threat and we felt that the concept of stratification applies in livestock as well as in orchards. The statistical methods described in this cited work are highly relevant to our experimental design and data analysis. We felt it would be unfair not to cite early work on sampling strategies for detection if the concepts are universal and have arisen from beyond our own discipline, no matter where they have been published.

**Reviewer 2:** *M&M*

*Pg. 8. In Austria PCR ran for 40 cycles, whereas in So. Africa, 35 cycles. In Table 2 the “target Ct is 15. It is unclear what constitutes a “positive” sample. By batching samples, the positive threshold needs to be well defined along with a measure (quality assessment) of the RNA extraction method such as an RNA host target of NADPH. How many PCR plate wells were included as duplicated samples to account for pipet errors/variations. This is a critical factor as the Ct is used for your mathematical (modeling) estimates?*

**Response:** A positive sample was determined through experimental design using a standard curve set up of three replicates of a known concentration of template. All quality checks were in place and a synthetic control was used to determine the exact concentration of points in the standard curve. Samples were tested in triplicate and mean Ct value determined to rule out pipetting errors. The Ct value used in the paper is a plausible value that corresponds to an unambiguously positive concentration of the ABSVd RNA in the plant.

**Reviewer 2:** *Pg. 9. Clarify “Potential patterning (privileged area)”.*

**Action:** We agree this would benefit from clarification and we have modified the text to read: “Potential patterning of infection within trees by which we mean the identification of areas that are consistently preferentially infected…”

**Reviewer 2:** *Pg10. Expt 3. It seems that mixing an extract from one ASBVd-positive leaf disk with 199 negative leaf disks is an extreme stretch of target PCR limits due to PCR inhibitors and random priming. Again, what is the criteria of a PCR-positive sample?*

**Response:** The aim of this experiment is not to set the PCR detection threshold, which we take as a given agreed by the regulatory authority, but to obtain an estimate for the slope (Â) of the relationship between the batch Ct and the dilution, which is required to optimise sampling protocol. For this purpose, having 200 leaves allowed us to study the relationship for a wide range of the dilution to estimate the slope reliably.

**Reviewer 2:** *Pg.10. Eq. 1. I understand Ctbatch but not Ctindividual for* *large number of samples. Clarify S1 Section C did not help make this clear.*

**Response:** The sample size is denoted n, Ct_i is the Ct for the unique infected leaf in the batch and Ct_batch is the batch Ct. Equation (1) gives the relationship between these three quantities.

**Reviewer 2:** *Pg.13. I was unable to open the “app”.*

**Response:** We are sorry the reviewer was unable to open the app. We can only think this was due to a temporary or internal technical issue. All co-authors have checked with colleagues in their respective institutes that the application is accessible and we have had positive feedback. The application is available and running at https://danielbonnery.shinyapps.io/ASBVd_Sampling/ (reference 22 of the manuscript). The app does not require any installation other than a web browser as it is available online and can be played when the webpage is launched.

**Reviewer 2:** *Pg.12-20. My mathematical expertise is limited so won’t comment on this section.*

**Response:** Noted: we are confident that the statistical methods are appropriate and correctly applied.

**Reviewer *2:*** *Discussion*

*Pg 20-23. As mentioned above, my mathematical expertise is limited so I can’t fairly evaluate the results. However,* *it seems the hierarchical bulk sampling portion would benefit by using a real-life field rather than only present results from modeling. For example, how many samples from trees can you safely batch to be able to discern a ASBVd-free orchard from one that has 1, 5, 10% infection rate? Seems this protocol has a pretty good change of missing an occasional ASBVd-infected tree.*

**Response:** The app specifically allows computation of the number of trees and leaves required in a sample to detect the viroid for a given value of the prevalence and a certain confidence level. When, for example, the regulatory requirements specify a PCR protocol for which we get $\hat{A}=4$, $\sigma=1$ and $Ct_{\left\{ \det ect \right\}}=25$, and specify$Ct_{\left\{ t\arg et \right\}}$=13, $\beta=0.67$, $\alpha=0.95$, then for a field of size 5000 and a prevalence of 1, 5, or 10%, the minimal number of trees to sample are: 299, 59 and 29 trees. We specifically put such real-life examples in Tables 2 and 3 to illustrate what the optimisation algorithm produces.

**Reviewer *2:*** *Moreover, I am not convinced of root-to-root transmission of ASBVd. In the case of other viroids of which I am familiar, mechanical transmission with pruning or cultivating equipment can be an efficient method of viroid spread from an inoculum source. I could not tell if this was accounted for in the dynamics of the model presented.*

**Response:** What matters for the accuracy of the statistical protocol to determine pest free status is not the precise nature of the transmission but whether the disease is likely to occur in clusters or to be randomly distributed in an orchard. We show in the simulations in the Supporting Information that systematic sampling can effectively account for each of these scenarios, while maintaining the risk of no detection for a given set of regulatory criteria.

***Reviewer 2:*** *Methods used to* *establishing a pest-free status for trade, indeed, needs a robust protocol and I am not able to judge how effective the methods described achieves this.*

**Response:** Through our paper and the app we aim to present and clarify to interested parties, the robustness of the new protocol. First, we identified the parameters that need to be set by the regulatory authorities and second, we provide an algorithm that optimizes the sample size. We demonstrate how multiple scenarios can produce reliable results using a user-friendly decision tool for use beyond academia.
